# Economic Analysis of Sequestering Carbon in Green Ash Forests in the Lower Mississippi River Valley

**DOI:** 10.1100/tsw.2003.61

**Published:** 2003-08-19

**Authors:** Ching-Hsun Huang, Gary D. Kronrad, Shiaolin D. Cheng

**Affiliations:** Arthur Temple College of Forestry, Stephen F. Austin State University, Box 6109, SFA Station, Nacogdoches, TX 75962-6109, USA

**Keywords:** carbon sequestration, Lower Mississippi Alluvial Valley, forest management

## Abstract

Since the U.S. is the largest emitter of carbon dioxide (CO_2_), it has become crucial to develop options that are both cost effective and supportive of sustainable development to reduce atmospheric CO_2_. Electric utility companies have the options of reducing their use of fossil fuels, switching to alternative energy sources, increasing efficiency, or offsetting carbon emissions. This study determined the cost and profitability of sequestering carbon in green ash plantations, and the number of tons of carbon that can be sequestered. The profitability of green ash is $2,342 and $3,645 per acre on site indices (measurement of soil quality) 65 and 105 land, respectively, calculated with a 2.5% alternative rate of return (ARR). These figures shift to –$248 and –$240 calculated with a 15.0% ARR. If landowners who have an ARR of 2.5% can sell carbon credits for $10 per ton of carbon, profits will increase by $107 per acre on poor sites and $242 on good sites. Over one rotation (cutting cycle), 38.56 net tons of carbon can be sequestered on an acre of poor quality land and 51.35 tons on good quality land. The cost of sequestering carbon, without including revenues from timber production and carbon credits, ranges from a high of $15.20 per ton on poor sites to $14.41 on good sites, calculated with a 2.5% ARR; to a high of $8.51 per ton on poor sites to $7.63 on good sites, calculated with a 15.0% ARR. The cost of storing carbon can be reduced significantly if the trees can be sold for wood products.

## INTRODUCTION

A leading environmental challenge of the 21st century will be to address the risks associated with global warming resulting from the long-term accumulation of greenhouse gases (GHG) in the atmosphere[1]. Strategies to reduce greenhouse gas emissions through innovative public- and private-sector voluntary initiatives are being developed. Among the best strategies will be low-cost and profitable opportunities for reducing greenhouse gas emissions. Capturing and storing carbon in forest plantations has been recommended as an economically and environmentally sound alternative for removing $CO_2$ from the atmosphere. Depending on location, establishment costs for forest plantations range from $93 to slightly more than $405 per acre with a median around $162[2]. Sedjo[3] predicted that the annual increase of atmospheric carbon is estimated to be 2.9 billion tons worldwide, and that it would require 1.1 billion acres of plantations at a cost of $372 billion (temperate zone) or $186 billion (tropics) to sequester this amount of carbon. Moulton and Richards[4] estimated that the costs of carbon sequestration range from $5.26 to $43.33 per short ton of carbon based on the direct social costs (the sum of the full cost of establishing trees and the market rental value of the land) over a period of 40 years. Plantinga et al.[5] used econometric land use models to estimate the costs of carbon sequestration in afforested stands in Maine, South Carolina, and Wisconsin. They found that marginal costs per ton of carbon rise from $0 to between $86 and $109 in Maine, $41 and $82 in South Carolina, and $68 and $86 in Wisconsin. The average cost estimates are approximately $54 per ton in Maine, $41 per ton in South Carolina, and $44 per ton in Wisconsin.

By contrast, according to the Carbon Sequestration Program Plans of the U.S. Federal Energy Technology Center, current cost estimates of sequestering carbon using present technologies range from $100 to $300 per ton of carbon emissions avoided[6]. Therefore, the U.S. Department of Energy (DOE) has established the long-term goal of reducing the cost of carbon sequestration to $10 or less per ton by 2015. If this goal is achieved, it will add less than one cent per kilowatt hour to the average electric bill[7] and make sequestration one of the most affordable options for addressing global warming.

The Lower Mississippi Alluvial Valley (LMAV), also described as the Delta, is one of the largest watersheds in the world with rich alluvial soils that received periodic sediment additions from the world's third largest river. The LMAV once supported highly productive ecosystems and the largest expanse of forested wetlands in the U.S.[8]. The combination of the Delta's rich soils, long growing season, and high rainfall resulted in rapid growth rates and high productivity of the region's bottomland hardwood forests[9]. Even though the LMAV had always been recognized for its agricultural potential, this area was susceptible to regular flooding for long durations, not only in late winter and spring but also in flash events during the summer[9]. The conversion of the forests was only economically feasible on the better-drained, higher elevation sites that could produce crops reliably enough[9]. This inhibited large-scale conversion of the forests to agricultural use. However, when the price of soybeans soared during the early 1970s, bottomland hardwood forests that once flourished on the tributaries of the lower Mississippi River and on its geological floodplain were cleared. Due to conversion to agriculture, the loss of bottomland hardwood forests in the LMAV approached 3.5 million acres[10]. Afforestation efforts have been made over the last 25 years. Through 1995, approximately 17,792 acres were afforested in the LMAV by acquisition of land by public agencies (i.e., U.S. Fish and Wildlife Service and U.S. Army Corps of Engineers) to enlarge federal wildlife refuges and to mitigate or offset wetland losses due to construction for flood control; 33,359 acres were afforested by state agencies (Mississippi, Louisiana, and Arkansas); 130,966 acres were afforested by Wetlands Reserve Program on private land[8]. It is projected that afforestation will increase, and by the year 2005, a total of 449,733 acres should be in afforestation schemes in the LMAV[8]. Since this will only account for a small percentage (13%) of the loss of bottomland hardwood forests in the

LMAV, a market for carbon sequestration credits might contribute to the financial feasibility of afforesting marginally productive agricultural land and encourage afforestation on a larger scale.

Reforestation of marginal or abandoned agricultural and pasture lands in the LMAV with bottomland hardwoods offers the greatest potential for significant net carbon storage in the South-Central Region of the U.S. Green ash (*Fraxinus pennsylvanica* Marsh.), the most widely distributed of all the American ashes, is a commercially valuable, fast-growing species that thrives on fertile, moist, well-drained bottomland sites[11]. Green ash is the most adaptable of all the ashes, growing naturally on a range of sites from clay soils subject to frequent flooding and overflow to sandy or silty soils where the amount of available moisture may be limited[12]. With a volume of 166.3 million cubic feet, green ash is the third most important commercial species in the Mississippi Delta region[13].

To some extent, a market for carbon credits already exists in the U.S. In a carbon credit market, electric utility companies will only invest in carbon sequestration projects if the cost is reasonable, and landowners will only grow forests to sequester carbon if it is profitable. Thus, evaluating the economic costs associated with the options that would mitigate the long-term increase in $CO_2$ becomes essential. It is also crucial to understand how a carbon credit market might work to motivate changes in silvicultural practices that would lead to increased carbon sequestration. Therefore, this study was designed to (1) calculate the profitability of managing forests for the dual products of timber and carbon storage, (2) calculate the total amount of carbon that can be stored by green ash trees grown on afforested marginal agricultural and unused pastureland in the LMAV, and (3) determine the average per ton cost (revenue) of sequestering carbon.

## METHODS

Forest Management Optimizer (FORMOP), a decision support system tool, was developed to determine the optimal number, timing, and intensity of thinning(s), and the optimal rotation (age at which mature timber is harvested), and to conduct cash flow analyses and calculate net present worth (NPW) and soil expectation values (SEV). FORMOP used the Forest Vegetation Simulator[14], a forest stand simulator, to predict stand growth data on diameter, height, and volume from establishment to final harvest for green ash. Site indices (the total height of the dominant trees at 50 years of age) of 65, 75, 85, 95, and 105 feet were used in the analyses. These site indices encompass the range of the most commonly observed, commercially acceptable soil qualities for green ash. The number of thinnings during the rotation could be zero, one, or two. Rotation length is the interval between one regeneration harvest and the next regeneration harvest. The first thinning could not be conducted until a green ash stand was at least 20 years of age. The minimum years between thinnings, or between a thinning and the final harvest, could not be less than 10 years. Four thinning intensities were employed: 20, 25, 30, or 35% of basal area removal. The same thinning intensities were used at all thinnings for a specific optimal solution regardless of the number of thinnings or age of thinning.

Six alternative rates of return (ARR), which span the range of before-tax earning rates available for most landowners, were chosen for the economic analyses. They were 2.5, 5.0, 7.5, 10.0, 12.5, and 15.0% in real terms, meaning that inflation has been removed. The annual real rate of price increase for green ash sawtimber and pulpwood were assumed to be 2 and 4.75%, respectively[15]. Labor costs were assumed to increase at a real rate of 1.12% per year[16]. The price of sawtimber was assumed to be $325 per thousand Doyle board feet, and pulpwood was priced at $16.50 per cord. It was assumed that reasonable, usual, and proper forest management activities would be conducted. Generally, management costs are incurred for establishing, maintaining, and harvesting the stand of trees. In this study, all current management costs came from a survey of forest consultants. Assumed management activities, frequency, and labor costs for green ash in the LMAV are presented in Table 1.

**TABLE 1**
**Management Operations and Labor Costs for Green Ash Plantations**

| Operations | Costs ($/acre) | Frequency | Start | End |
|---|---|---|---|---|
| Site preparation and Planting | | | | |
| Seedling | $114 | Once only | Year 0 | |
| Planting | $68 | Once only | Year 0 | |
| Subsoiling | $10 | Once only | Year 0 | |
| Herbicide | $40 | Once only | Year 0 | |
| Management Plan | | | | |
| Initial Plan | $5 | Once only | Year 0 | |
| Update Plans | $10 | Every 10 years | Year 10 | Final |
| Boundary Maintenance | | | | |
| Initial | $10 | Once only | Year 0 | |
| Update | $2 | Every 10 years | Year 10 | Final |
| Mark and administer timber sale | 10% of timber revenues | -AS NECESSARY- | | |

It was assumed that a market would develop in which private companies, needing to offset their carbon emissions, would pay landowners for each additional ton of carbon that they sequester in their forests. Landowners would want to maximize the net revenue from the production of three products: sawtimber, pulpwood, and tons of carbon. Sawtimber and pulpwood have market prices that are readily determined. Carbon, on the other hand, presently is not a tradable commodity with a market price in the U.S. Therefore, in these analyses, the price (or value) of carbon was assumed to be $10, $50, or $100 for each additional ton of carbon that landowners sequester in their green ash plantation. This assumed price range would encompass most carbon credit prices predicted for the future carbon credit market. Economic analyses for timber production management only ($0 carbon value) were also conducted to produce baseline data.

Carbon stored in the wood products, soils, and forest floor was calculated. For the above-ground tree biomass, only carbon stored in the useable portion of pulpwood or sawtimber qualified as carbon credits. In order to estimate the amount of carbon stored in trees, the dry weight equation for green ash in the west-central Mississippi[17] was applied to pulpwood and sawtimber. It was then assumed that the roots of green ash account for 19.7% of the total (above- plus below-ground) tree biomass[18]. Because net amount of carbon in trees is estimated to be 50% of dry biomass[19], the estimated amount of carbon was determined by multiplying the tree dry weight by 50%. Estimates of organic soil carbon and carbon on the forest floor were derived from a study conducted by Birdsey[20].

It was assumed that as trees grow larger and store more carbon, landowners would receive an annual payment based on the amount of carbon sequestered and the price of carbon. When a stand's mortality is greater than its growth, or a thinning or final harvest was conducted, landowners would repay the carbon credit buyers for the loss of tree biomass in which the carbon was stored. This repayment was calculated based on how many tons of carbon were lost from the stand and how much each ton of carbon was worth. No repayment was required for wood used to produce long-lived wood products. Since they continue to sequester carbon, the lifetime of these timber products as it relates to carbon sequestration was assumed to be over multiple rotations. All financial gains and losses from carbon sequestration within the rotation were included in the discounted cash flow analyses.

Given a range of site indices, real ARRs, and carbon prices, discounted cash flow analyses were conducted to obtain NPW for all the operable management regimes. The Faustmann formula was then applied to calculate SEV. The management regime that had the highest SEV was chosen as the financially optimal "thinning and final harvest" schedule for each combination of site index and landowner's ARR.

## RESULTS

This study investigated the efficiencies and effects of storing carbon in forests and establishing a carbon credit market from the viewpoint of both carbon credit buyers (electric utility companies) and carbon credit sellers (forest landowners). From the buyers' perspective, before they will invest in forest management to sequester carbon or pay landowners to grow trees for them, they will want to know: (1) How much carbon can be stored in a forest? and (2) How much does it cost to store a ton of carbon? A total of 75,210 operable thinning and harvesting combinations and cash flow analyses, including soil expectation values, were calculated. Table 2 presents the number of tons of carbon that can be sequestered on one acre of green ash plantation, given a range of ARRs and site indices. Site index (or soil quality) and the ARR (or interest rate) affect the timing, number, and intensity of the thinnings, and the timing of the final harvest. It should be noted that, in general, to maximize financial return, as the ARR increases, the rotation length decreases. And as the site index increases, the rotation length decreases. This, in turn, affects the number of tons of carbon that are sequestered during one rotation. The number of tons of carbon that may be sequestered ranges from a low of 29.55, calculated on site index 65 land using an ARR of 15%, to a high of 51.35 tons, calculated on site index 105 land using an ARR of 2.5%. Tonnage presented in this table is the net amount of carbon stored throughout one rotation.

**TABLE 2**
**Tons of Carbon Stored per Acre using the Optimal Thinning and Harvesting Schedule for Green Ash Plantations, by Site Index and Real ARR**

| | Real ARR (%) | | | | | |
|---|---|---|---|---|---|---|
| **Site Index** | **2.5** | **5.0** | **7.5** | **10.0** | **12.5** | **15.0** |
| 65 | 38.56 | 38.56 | 38.30 | 34.21 | 30.74 | 29.55 |
| 75 | 38.63 | 40.76 | 34.49 | 30.95 | 31.56 | 30.24 |
| 85 | 46.26 | 42.80 | 41.99 | 36.70 | 32.70 | 31.18 |
| 95 | 48.52 | 44.86 | 44.21 | 37.88 | 32.79 | 32.79 |
| 105 | 51.35 | 47.03 | 46.55 | 39.57 | 34.91 | 33.08 |

Table 3 presents the cost to store a ton of carbon on an acre of land planted with green ash, given a range of ARR and site indices. This calculation was performed by dividing the present value of all forest management costs by the total number of tons of carbon stored. The cost to store a ton of carbon ranges from a low of $6.05, calculated on site index 105 land using an ARR of 7.5%, to a high of $16.46, calculated on site index 75 land using an ARR of 2.5%. For those market-competitive interest rates of 5.0 to 15%, the cost of sequestering carbon in green ash forests is below the U.S. Department of Energy's long-term cost goal of sequestering carbon for $10 or less per ton by 2015. Yet, this kind of cost analysis may be misleading. As trees grow, they not only store carbon but also produce marketable wood products. Properly managing forestland for timber production can be very profitable. Sequestering carbon in forests grown for timber production often requires no additional management costs. Therefore, if timber production is profitable, carbon sequestration is just a positive externality of profitable and proper timber management, and carbon sequestration is costless. Table 4 presents the amount of money earned by selling each ton of carbon as a timber product. It was calculated by dividing present value of net revenue earned from timber management by the total number of tons of carbon stored. For example, landowners who have a 2.5% ARR and own land with a site index of 105 would earn $70.73 for each ton of carbon stored by selling the timber products. Landowners who have a 15% ARR and site index 65 land, would loss $8.41 from the sale of timber products for each ton of carbon stored. Given that landowners will have timber products to sell regardless of the existence of carbon trading, Table 4 presents more accurate data on the true cost of carbon sequestration in green ash forests.

**TABLE 3**
**Cost ($) of Storing a Ton of Carbon using the Optimal Thinning and Harvesting Schedule for Green Ash Plantations, by Site Index and Real ARR**

| | Real ARR (%) | | | | | |
|---|---|---|---|---|---|---|
| **Site Index** | **2.5** | **5.0** | **7.5** | **10.0** | **12.5** | **15.0** |
| 65 | 15.20 | 8.41 | 7.08 | 7.54 | 8.25 | 8.51 |
| 75 | 16.46 | 8.13 | 7.94 | 8.37 | 8.05 | 8.32 |
| 85 | 14.49 | 7.90 | 6.58 | 7.08 | 7.78 | 8.08 |
| 95 | 14.52 | 7.70 | 6.30 | 6.88 | 7.77 | 7.68 |
| 105 | 14.41 | 7.50 | 6.05 | 6.61 | 7.31 | 7.63 |

**TABLE 4**
**Profits Earned ($) from Each Ton of Carbon Stored using the Optimal Thinning and Harvesting Schedule for Green Ash Plantations, by Site Index and Real ARR**

| | Real ARR (%) | | | | | |
|---|---|---|---|---|---|---|
| **Site Index** | **2.5** | **5.0** | **7.5** | **10.0** | **12.5** | **15.0** |
| 65 | 60.73 | 5.43 | –4.30 | –6.80 | –7.98 | –8.41 |
| 75 | 70.12 | 6.93 | –4.09 | –7.25 | –7.66 | –8.16 |
| 85 | 65.31 | 8.61 | –2.85 | –5.90 | –7.29 | –7.86 |
| 95 | 68.60 | 9.12 | –2.17 | –5.47 | –7.12 | –7.41 |
| 105 | 70.73 | 10.10 | –1.54 | –5.00 | –0.15 | –7.26 |

From the carbon credit sellers' point of view, before landowners will engage in forest management to sequester carbon and produce timber, they will want to know: (1) Is forest management for timber production profitable? and (2) Is management for timber and carbon credits profitable? Table 5 shows the profitability of managing green ash forests for timber production and carbon credits, given a range of site indices and ARRs. When carbon credits are valued at $0 per ton, for a landowner who has site index 65 land and whose best alternative investment earns 2.5%, he can earn $2,342 per acre by managing his land for green ash timber production. If he owns site index 105 land and has an ARR of 15%, he will earn $240 less than his ARR, in net present worth terms. Forest management for timber production only is profitable on site indices 65 through 105 using ARRs of 2.5 and 5.0%. Management is not profitable for ARRs of 7.5 through 15%.

**TABLE 5**
**Net Present Worth ($) per Acre with Timber Revenues and Carbon Credits (CC) using the Optimal Thinning and Harvesting Schedule, by Site Index and Real ARR**

| | Real ARR (%) | | | | | |
|---|---|---|---|---|---|---|
| | **2.5** | **5.0** | **7.5** | **10.0** | **12.5** | **15.0** |
| Site Index 65 | | | | | | |
| CC = $0/ton | 2,342 | 209 | –165 | –233 | –245 | –248 |
| CC = $10/ton | 2,449 | 264 | –138 | –219 | –237 | –243 |
| CC = $50/ton | 2,879 | 482 | –29 | –163 | –206 | –224 |
| CC = $100/ton | 3,417 | 755 | 107 | –93 | –167 | –200 |
| Site Index 75 | | | | | | |
| CC = $0/ton | 2,709 | 282 | –141 | –224 | –242 | –247 |
| CC = $10/ton | 2,848 | 352 | –105 | –205 | –230 | –239 |
| CC = $50/ton | 3,407 | 632 | 36 | –129 | –185 | –211 |
| CC = $100/ton | 4,106 | 982 | 214 | –33 | –129 | –175 |
| Site Index 85 | | | | | | |
| CC = $0/ton | 3,021 | 343 | –120 | –217 | –238 | –245 |
| CC = $10/ton | 3,197 | 426 | –73 | –191 | –223 | –235 |
| CC = $50/ton | 3,899 | 760 | 111 | –89 | –161 | –194 |

| | | | | | | |
|---|---|---|---|---|---|---|
| CC = $100/ton | 4,778 | 1,178 | 342 | 40 | –84 | –143 |
| Site Index 95 | | | | | | |
| CC = $0/ton | 3,329 | 409 | –96 | –207 | –233 | –243 |
| CC = $10/ton | 3,540 | 511 | –38 | –175 | –214 | –229 |
| CC = $50/ton | 4,384 | 916 | 194 | –43 | –134 | –173 |
| CC = $100/ton | 5,440 | 1,423 | 484 | 121 | –35 | –103 |
| Site Index 105 | | | | | | |
| CC = $0/ton | 3,645 | 475 | –72 | –198 | –229 | –240 |
| CC = $10/ton | 3,887 | 593 | –1 | –156 | –210 | –215 |
| CC = $50/ton | 4,857 | 1,066 | 284 | 9 | –134 | –114 |
| CC = $100/ton | 6,071 | 1,657 | 639 | 217 | 35 | –57 |

The profitability of forest management increases if carbon credits can be marketed. For example, if carbon credits can be sold for $10 per ton, the profitability of management on site index 95 land, 5.0% ARR, increases from $409 without carbon credits to $511 per acre with carbon credits (Table 5). For site index 105, ARR of 7.5%, profitability increases from a loss of $72 per acre without carbon credits to a loss of just $1 with the sale of carbon credits at $10 each. If this landowner can sell his carbon credits for $50 per ton he would earn his 7.5% return on investment plus an additional $284. And his profit would increase to $639 per acre if his carbon credits could be sold for $100 per ton.

## DISCUSSION

Properly utilizing forests can mitigate global warming by reducing greenhouse gas emissions while improving economic efficiency, increasing rural employment, and enhancing the environment. Afforestation of marginal agricultural land in the LMAV would rely on native species, planted mostly in single-species plantations[8]. For successful regeneration and afforestation, species preferences and tolerances must be matched to site variation within floodplains and site requirements of the species to be used[8]. Since the dominant, perennial species of the LMAV are limited to those that can tolerate the anaerobic stress associated with frequent, prolonged flooding during the growing season, plant species diversity tends to be moderate or low[9]. This shortens the species lists for these communities considerably, reducing their contribution to global biodiversity but, on the other hand, simplifying the task of restoring bottomland hardwood forests in the lower Mississippi Valley[9]. The predicted rising stumpage prices for hardwoods and changes in agricultural price supports might favor private afforestation on marginal farmland, particularly if economic incentives for carbon sequestration could be captured by landowners[21].

Green ash is a valuable bottomland hardwood species that tolerates periodic flooding and grows better than other species when available water is low. The results of this study indicate that when forest landowners' real ARR is low (2.5 or 5.0%), the profitability of managing green ash plantations is positive even when timber is the sole product. When the assumed carbon price is increased to $50 per ton, the profitability increases significantly. Although some uncertainty exists concerning the future for carbon credits, there seems to be agreement that afforestation will be eligible[22]. In the foreseeable future, a carbon credit market will be established in the U.S. The methods used in this study will help to investigate the impacts of timber product and carbon credit prices.
